# Making Chatbots more human: deep reasoning large language models in ophthalmology

**DOI:** 10.3389/fmed.2025.1741888

**Published:** 2026-01-12

**Authors:** Xuanqiao Lin, Yizhou Yang, Yuecheng Ren

**Affiliations:** 1 Department of Ophthalmology, Eye, Ear, Nose, and Throat Hospital of Fudan University, Shanghai, China; 2 Department of Ophthalmology, Jiaxing Traditional Chinese Medicine Hospital Affiliated to Zhejiang Chinese Medical University, Jiaxing, Zhejiang, China

**Keywords:** large language models, artificial intelligence, ophthalmology, clinical decision support, Chatbots, deep reasoning

## Abstract

Recent advances in deep-reasoning large language models (LLMs)—including OpenAI's GPT series and open-source DeepSeek models—have expanded their potential applications in ophthalmology. In ophthalmology, image interpretation continues to rely primarily on conventional computer vision and vision language model pipelines, whereas text-based LLMs contribute to language-centric workflows, such as report interpretation, patient education drafting, and electronic health record (EHR) summarization. Multimodal systems that integrate visual inputs with reasoning have been explored in simulated or retrospective settings for tasks such as personalized planning. Although these approaches may enhance workflow efficiency and decision-making, their direct clinical benefits have not yet been established. Nevertheless, practical implementation remains challenging because of computational demands, privacy and bias considerations, and persistent issues with transparency and interpretability. Additionally, system congestion and inconsistent response times further complicate real-world clinical use. Therefore, future research should focus on addressing operational and ethical constraints, tailoring AI systems to ophthalmic workflows, and ensuring that such tools remain an assistive, equitable, and transparent partner in clinical decision-making. Thoughtful integration of deep reasoning models appears promising for ophthalmic practice, but prospective interventional studies are required before making any claims regarding patient outcomes.

## 1 Introduction

With recent advancements in deep learning (DL)—particularly the development of transformer-based models such as BERT and GPT—large language models (LLMs) have achieved remarkable accuracy in domains including natural language processing and image recognition (1, 2). Although they demonstrate impressive capabilities, conventional LLMs often struggle with handling tasks that require deeper reasoning and deliberate, multi-step logic (Table 1).

**Table 1:** Comparison between new large language models and traditional artificial intelligence models.

**Comparison dimension**	**Deep reasoning LLMs**	**LLMs**	**Traditional AI models**
Parameter scale	Typically hundreds of billions, with structured reasoning modules	Hundreds of billions to tens of trillions	Thousands to millions
Architecture	Enhanced transformer with GNNs, reasoning chains	Transformer with deep self-attention	Simple structures, e.g., SVM, shallow RNNs
Training method	Multi-step thinking and chain-of-thought fine-tuning	Pre-training + fine-tuning, self-supervised learning	Trained from scratch, relies on labeled data
Task scope	Reasoning, QA, multi-hop, planning, etc.	Discriminative + generative, multi-task supported	Single-task: classification, prediction, clustering
Performance	Much improved on complex logical reasoning	Strong on complex tasks, good generalization	Efficient on simple tasks, weak generalization
Computational resources	Extremely high resource usage, requires clusters or specialized chips	Requires high-performance hardware, high energy consumption	Runs on standard hardware, low energy consumption
Interpretability	Improved via explicit reasoning paths	Limited interpretability	High interpretability
Training data	Augmented with structured data (graphs, rule bases)	Large-scale, diverse text data	Small, labeled, task-specific datasets

LLM, large language models; AI, artificial intelligence; GNNs, graph neural networks; QA, question answering; SVM, support vector machines; RNNs, recurrent neural networks.

In ophthalmology, the rapid progress of LLMs has primarily led to applications in language-centered workflows, such as interpretation of narrative clinical reports, generation of patient education materials, summarization of electronic health record (EHR) notes, and supporting referral triage (3, 4). More recently, frontier multimodal models that incorporate visual processing have begun to provide workflow-level assistance—for example, structuring biometry data for intraocular lens (IOL) calculations or interpreting the spatial layout of complete visual field test reports (5, 6).

Within this review, the capability for Deep Reasoning is defined as a model capable of explicit, multistep decomposition, verification, and self-correction during the reasoning process and improving the stability of its conclusions through leveraging test-time compute or self-consistency when necessary (7). Deep Reasoning does not mean “larger models”; it refers to a structured reasoning workflow that has systematically mitigated common failure modes of traditional LLMs in clinical reasoning, including hallucinations, cross-step logical breaks, and inconsistent conclusions (8).

Advances in reasoning-oriented LLMs are particularly relevant to ophthalmology. These models employ mechanisms such as extended chain-of-thought (CoT), test-time scaling, and reinforcement learning from human feedback (RLHF) to improve logical consistency, internal verification, and iterative refinement (9). Such capabilities create opportunities for enhancing diagnostic reasoning and individualized patient counseling in both benchmarked and simulated settings. However, translating these systems into clinical practice remains challenging due to issues related to computational efficiency, ethical safeguards, and model interpretability (4).

Ophthalmology is particularly suitable for deep-reasoning LLMs because most ophthalmic conditions require longitudinal decision-making and multi-step therapeutic planning rather than one-off answers (10, 11). Disease management often proceeds across repeated visits, necessitating an integration of symptoms, imaging-derived reports, refraction, intraocular pressure, comorbidities, and treatment responses (12). Clinicians use this evolving information to update differential diagnoses, refine risk stratification, and adjust stepwise care pathways. Such workflows demand multi-step reasoning, consistency checks, and plan verification, matching the core strengths of deep-reasoning LLMs compared with conventional single-pass generation.

This review provides a comprehensive overview of the evolution of LLMs, highlights the emergence of deep-reasoning architectures, and examines their current and potential applications in ophthalmology. It also discusses major limitations and outlines future research priorities essential for safe and effective integration of these technologies into clinical practice.

## 2 Method

Following PRISMA-aligned best practices for narrative reviews, we searched PubMed and Web of Science Core Collection using the query (ophthalmology OR eye) AND (“large language model” OR LLM OR “vision language model” OR “chatbot”) AND (diagnos^*^ OR triage OR report^*^ OR “IOL” OR surgery OR “instrument tracking” OR “decision support”) with a publication window from 1980 to 2025. This search identified 251 unique records in PubMed and 215 in Web of Science. After cross-database deduplication, 357 remained. Title screening retained 159 records and excluded 198, while abstract screening retained 99 and excluded 60. The most common reasons for exclusion during abstract screening were lack of an available abstract (*n* = 33), the task falling outside the scope of the review (*n* = 21), and reviews lacking empirical evaluation (*n* = 6). We compiled an inclusion list with the following source-specific identifiers: PMID or UT, publication year, and article title. Consistent with the narrative review, we did not register a review protocol or apply a formal risk of bias assessment tool.

## 3 Development and current status of deep reasoning LLMs

### 3.1 Development of deep reasoning LLMs

The advent of deep reasoning LLMs can be traced to the introduction of transformer architectures in natural language processing (NLP). Introduced in 2017, the Transformer model replaced traditional recurrent neural networks (RNNs) with an attention mechanism, substantially enhancing sequence data processing capabilities (13). OpenAI's GPT series, launched in 2018, further advanced LLM development through a pretraining-fine-tuning framework (14). With GPT-3 demonstrating significant emergent reasoning abilities in 2020, although limitations in complex, multi-step reasoning tasks remained (15).

By 2022, studies showed that prompting models to generate intermediate reasoning steps—known as chain-of-thought (CoT)—significantly improved performance on complex reasoning benchmarks (16). Techniques, such as self-consistency, which involved sampling multiple reasoning paths and selecting a consensus answer, further increased accuracy (17). More recently, reinforcement learning has been introduced to encourage coherent reasoning traces. Although these techniques enhance benchmark performance, their contribution to genuine reasoning remains under debate. Recent analyses question whether CoT reliably scales to high-complexity or out-of-distribution problems (18). Consistent with this view, a more rigorous framing characterizes current models as strong in crystallized intelligence—the recall and application of accumulated knowledge—yet still limited in fluid intelligence, which requires adaptive and flexible problem solving.

Concurrently, the field has shifted toward a slow-thinking paradigm that integrates training-time optimization with test-time computation strategies. Test-time scaling, structured multistep reasoning, and verification are increasingly combined with reinforcement learning from human or programmatic feedback to improve the reliability and consistency of reasoning processes. This integration is often described as a pathway toward more robust stepwise deliberation, although it still faces challenges in terms of efficiency, interpretability, and generalization (4).

Beyond text-based approaches, hybrid pipelines have emerged in which structured or relational models complement LLMs. Graph neural networks (GNNs) can capture structural dependencies, and when integrated with convolutional neural network (CNN) feature extractors, they enable tasks that combine imaging with language-based reasoning. Such modular integrations do not necessarily make the LLM itself multimodal; rather, they illustrate a system-level design in which visual features inform downstream reasoning for diagnostic and imaging analysis scenarios (19).

To avoid conflating different technical routes, this review classify ophthalmic “image interpretation” systems into three categories: (1) traditional computer-vision models, which process images alone for tasks such as classification or segmentation; (2) native vision–language models, which take multimodal inputs of images and text and conduct unified reasoning across vision and language; and (3) text LLMs coupled with external visual encoders, where image information is first converted to structured descriptors in the form of features, captions, or reports, and subsequently reasoned over in text. Thus, if a deep reasoning LLM does not take native image input, such as DeepSeek-R1, its primary clinical value should be framed in terms of superior textual reasoning—for example, interpreting ophthalmic reports, operative notes, and longitudinal records—rather than direct image reading.

### 3.2 Representative deep reasoning LLMs

#### 3.2.1 GPT-o1

In 2024, OpenAI introduced GPT-o1, a model specifically engineered for deep reasoning. Unlike previous LLMs, GPT-o1 generates extended sequences of intermediate reasoning steps before producing a final response, thereby strengthening its ability to manage complex, multi-step logical tasks. Using reinforcement learning, particularly RLHF, GPT-o1 was trained to refine these intermediate reasoning processes, which improved its benchmark performance on multistep tasks. GPT-o1 has also demonstrated strong performance in mathematics competitions such as the American Invitational Mathematics Examination (AIME) and programming challenges.

In medical imaging analysis, GPT-o1's ability to process visual input and leverage internal reasoning processes has the potential to improve diagnostic accuracy. Recent studies have shown that GPT-o1 achieves diagnostic accuracies comparable to those of professional clinicians in clinical case analyses (20). However, the computational intensity and associated costs of GPT-o1 pose practical challenges for its widespread clinical implementation (20).

#### 3.2.2 ChatGPT 5

In August 2025, OpenAI introduced GPT-5, a unified system featuring two complementary modes—a fast default mode and a dedicated thinking mode—automatically selected by a routing mechanism according to task complexity and user requirements. This design allows the adaptive allocation of computational resources and effectively balances efficiency with reasoning depth. GPT-5 achieved 94.6% accuracy on the AIME-2025 benchmark for mathematical reasoning and set new state-of-the-art results on SWE-bench Verified, MMMU, and HealthBench-Hard. Additionally, it demonstrated improvements in instruction adherence, reduced hallucination rates, and greater robustness against sycophantic responses (21).

In biomedical and clinical contexts, GPT-5 functions as an active thought partner rather than a decision-maker, showing cautious reasoning behavior and dynamic contextual adaptation. On the MedXpertQA-MM benchmark, GPT-5 outperformed GPT-4o by +29.26% in reasoning and +26.18% in comprehension performance, exceeding the scores of licensed human experts by +24.23 and +29.40%, respectively (22). Despite these promising outcomes, GPT-5 remains a newly released model, and its broader implications for medical diagnosis and clinical education warrant further systematic evaluation.

#### 3.2.3 Deepseek-R1

In early 2025, the open-source community introduced DeepSeek-R1, a large language model designed to advance deep reasoning through reinforcement learning (RL). Unlike conventionally supervised models, DeepSeek-R1 employs a multi-stage optimization pipeline that integrates “cold-start” supervised fine-tuning, rule-based RL, rejection sampling, and an additional RL phase incorporating preference and safety rewards (23). This approach enhances the coherence and interpretability of reasoning processes while reducing computational costs to ~5%−10% of those required by GPT-o1, yet maintaining comparable performance on complex reasoning benchmarks (24).

A defining feature of DeepSeek-R1 is its emphasis on the chain-of-thought (CoT), which enables systematic problem analysis and synthesis. Notably, emergent behaviors such as self-reflection and verification have been observed during training. Furthermore, the model's reasoning capability has been successfully distilled into smaller variants, increasing accessibility for research and clinical developers (23). Its performance on medical reasoning tasks, including ophthalmic case studies, matches proprietary models, with an accuracy of ~82% (24). Because of its open-source nature, it supports fine-tuning tailored to specific medical domains. Although it does not natively support multimodal inputs, DeepSeek-R1 can be integrated with external vision encoders (e.g., CNN-based feature extractors) to enable language–image workflows in medical imaging analysis, thereby providing a flexible and cost-efficient platform for both research and clinical applications. Reported limitations include suboptimal structured output and tool use, reduced token efficiency issues on simple queries, prompt sensitivity (with zero-shot prompting preferred), and prior language-mixing issues that have since been mitigated through a language-consistency reward mechanism (23).

#### 3.2.4 Grok-3

In early 2025, xAI introduced Grok-3, an advanced LLM designed with enhanced reasoning capabilities, supported by specialized inference methods like “Think Mode” and “Big Brain Mode,” and capable of more in-depth analysis and nuanced decision-making. Grok-3 performed well, achieving competitive scores on complex reasoning tasks, including a high score from the 2025 AIME (25). In the biomedical context, Grok-3 shows promising potential applications in ophthalmology, such as enhancing diagnostic accuracy, facilitating personalized treatment planning, and supporting patient education.

### 3.3 Additional research in deep reasoning LLMs

In addition to GPT-o1 and Deepseek-R1, other institutions have explored alternative strategies for enhancing LLM reasoning abilities. Anthropic's Claude series utilizes extended context windows to improve the handling of long-chain reasoning tasks (26). Google's Gemini series explores dynamic allocation of computational resources during reasoning—often referred to as test-time compute—to enhance performance on complex tasks. Furthermore, ongoing research focuses on integrating external knowledge bases and computational tools, such as code execution, to further optimize LLM reasoning capabilities and highlight potential future developments.

## 4 Application of deep reasoning models in ophthalmology

### 4.1 Diagnostic assistance

Previous studies have demonstrated that LLMs achieve high diagnostic accuracy in ophthalmology (27, 28). Deep reasoning models—such as DeepSeek-R1, GPT-o1, Gemini 2.0, and Grok3—have shown significant potential in ophthalmic diagnostics, particularly for conditions such as glaucoma, diabetic retinopathy (DR), and age-related macular degeneration (AMD) (29, 30). Notably, most of these evaluations were conducted in retrospective, benchmark, or simulated settings, and thus should be interpreted as performance evidence rather than demonstrated real-world patient outcome benefit. By analyzing large volumes of medical data, including symptoms, test results, and medical history, these models can provide doctors with diagnostic recommendations and differential diagnosis lists (31). In principle, this capability may reduce the risk of diagnostic errors and assist ophthalmologists in making more informed clinical decisions. A comparative evaluation of DeepSeek-R1 and GPT-o1 in pediatric clinical decision support reported diagnostic accuracies comparable to those of clinical specialists (92.8% for GPT-o1 and 87.0% for DeepSeek-R1), underscoring their potential as clinical decision support tools (32).

Beyond ophthalmology, LLMs have also demonstrated high accuracy in automated extraction of Coronary Artery Disease-Reporting and Data System (CAD-RADS) 2.0, components from semi-structured coronary CT angiography reports, particularly when used with CoT prompting (33). These findings underscore the broader value of structured reasoning strategies in improving reliability across clinical domains and further support their potential utility in ophthalmology.

**Figure 1:**
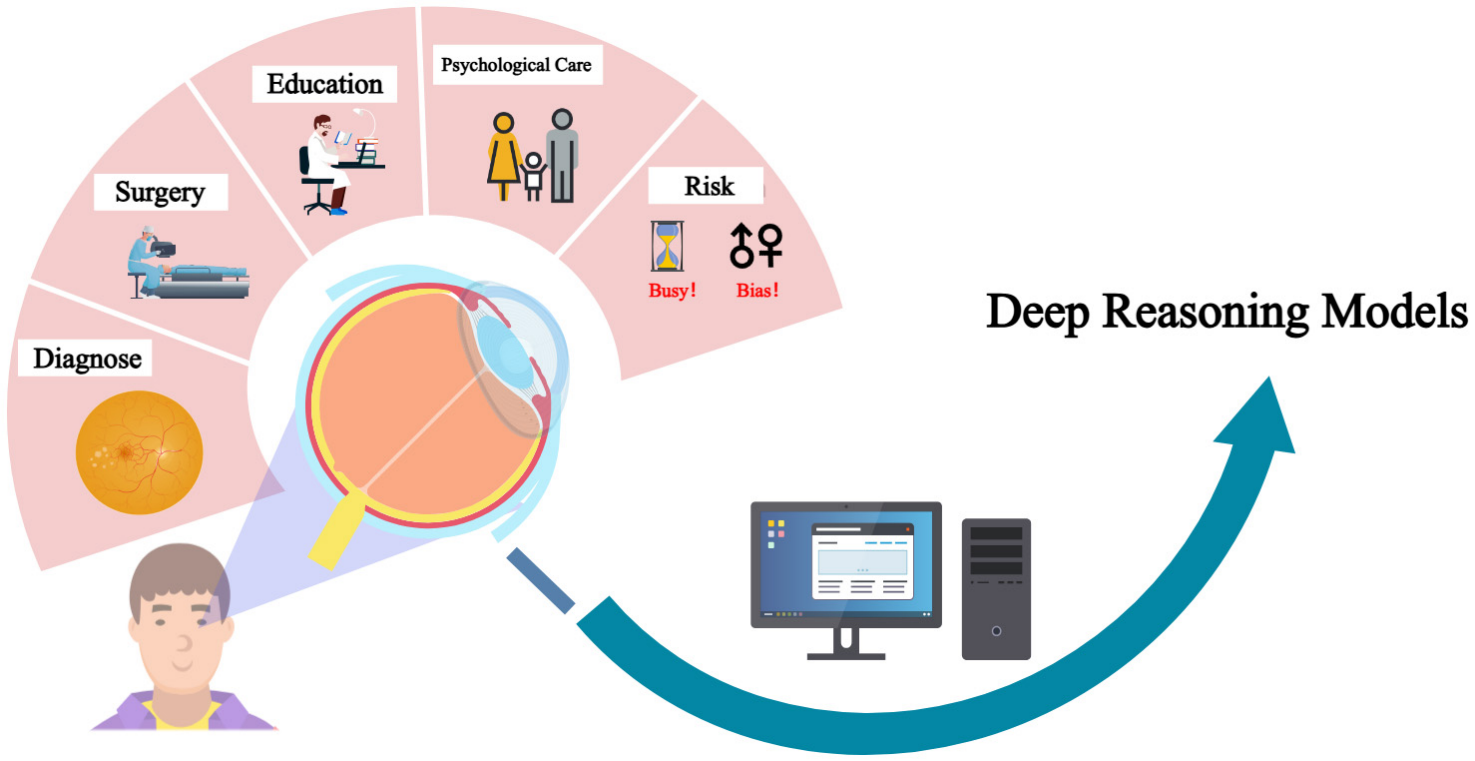
Application and risk of deep reasoning models in ophthalmology.

Ophthalmology is uniquely positioned for the integration of deep-reasoning LLMs due to the abundance of quantifiable ocular biometric parameters, such as axial length, anterior chamber depth, lens thickness, and corneal curvature, routinely obtained through imaging modalities including OCT, B-scan ultrasonography, and anterior segment photography. This extensive repository of structured and image-derived data provides a strong foundation for applying reasoning models to diagnostic and analytical workflows. However, some limitations remain because not all deep-reasoning models support multimodal learning. For instance, DeepSeek-R1 remains confined to text-based inputs and cannot natively process images. However, its open-source framework enables customized server configurations that may partially mitigate this limitation and extend its applicability to ophthalmology (Figure 1).

### 4.2 Surgical assistance

Traditional LLMs have been explored for perioperative support in both retrospective and simulation settings (34, 35). Through improvements in preoperative planning and postoperative monitoring, deep reasoning models are progressively influencing ophthalmic surgical procedures. Most deep-reasoning LLMs, with their transparent reasoning pathways, can provide explicit justifications for surgical decision-making, thereby enhancing clinician confidence (6). LLMs are capable of interpreting IOLMaster outputs and assisting with the selection of toric or non-toric IOLs, although the power calculation itself continues to rely on conventional statistical or machine learning formulas (5). Consequently, the LLM contribution is more accurately described as IOL selection support rather than IOL power calculation, to clearly distinguish language-model assistance from formula-based computation. Moreover, in the future, multimodal pipelines that couple external visual encoders with text-based DR-LLMs could support intraoperative decision, further augmenting surgeons' capabilities and enhancing patient safety. Postoperatively, AI systems facilitate remote wound-healing assessment, early identification of complications, and improved recovery outcomes.

### 4.3 Patient education

Deep-reasoning LLMs can enhance patient management by providing personalized therapeutic recommendations, educational content, and remote monitoring support. These models effectively simplify complex medical information, making it more understandable and engaging for patients, which in turn improves comprehension and adherence to ophthalmic treatments. For example, they can generate easy-to-understand explanations of medical conditions, treatments, and procedures to help patients understand their health better and make informed decisions. They can also create personalized educational materials based on the specific needs and understanding levels of patients, thereby enhancing the effectiveness of patient education (36, 37).

Several comparative studies have examined the role of different LLMs in patient education and demonstrated promising results (38, 39). Some models have achieved better readability and comprehensibility than those provided by clinicians. Semeraro et al. (32) evaluated models such as ChatGPT-4o, Gemini Advanced, and DeepSeek-R1 for their ability to convey critical medical guidelines—such as those from the European Resuscitation Council (ERC) on CPR—to the public. These studies showed promising results in terms of readability and correctness metrics for patient education.

### 4.4 Patients' psychological care

A recent study examined the ability of six LLMs—GPT-4o, GPT-o1, DeepSeek-R1, Claude 3.5 Sonnet, Sonar Large (LLaMA-3.1), and Gemma-2-2b—to detect risks of domestic violence, suicide, and filicide suicide within the Taiwanese flash fiction *Barbecue* (40). Notably, GPT-o1 demonstrated an ability to identify suicide risk based on subtle cultural cues, suggesting that deep reasoning–based LLMs may outperform traditional models in recognizing latent psychological states during conversations. This observation holds particular significance in ophthalmology, where patients with ocular trauma, advanced glaucoma, or optic nerve atrophy often experience irreversible vision loss and poor surgical outcomes, which may lead to despair, resentment, or even suicidal ideation (41, 42).

However, recent reports such as Chatbot psychosis have highlighted that the unconstrained conversational use of LLMs in psychiatric contexts may inadvertently exacerbate symptoms or undermine the clinician–patient relationship (43). In light of these concerns, future applications should avoid allowing LLMs to provide unsupervised psychiatric counseling. Instead, more restricted formats—such as one-way question-answer screening tools or non-dialogue-based linguistic analyses—could enable the early recognition of at-risk patients while mitigating the psychological risks associated with unrestricted conversational interactions. Thus, although deep-reasoning LLMs show considerable promise in identifying latent psychological distress, their role must be carefully designed with strict ethical oversight.

### 4.5 Evidence gap: performance vs. clinical benefit

Although deep-reasoning LLMs show promising accuracy and workflow performance in benchmarks, retrospective analyses, and simulated scenarios, direct clinical benefits have not yet been established. Importantly, recent benchmark research in medicine has highlighted a broader shift from knowledge-based testing (where leading models may reach near-saturated performance) to practice-based assessment, revealing a substantial knowledge–practice gap in which high examination scores can be misleading proxies for clinical readiness and safety (44). Accordingly, future work in ophthalmology should prioritize prospective interventional studies and practice-oriented validation that evaluate clinically meaningful endpoints beyond accuracy, such as reductions in diagnostic errors, time-to-decision, unnecessary testing/referrals, and downstream visual outcomes (e.g., postoperative refractive error, visual acuity, complication rates), as well as patient-centered outcomes, ideally within powerful human oversight frameworks.

## 5 Risks and challenges in clinical implementation

Despite these promising developments, deep-reasoning LLMs face several significant challenges. Models such as Deepseek-R1 and GPT-o1 require considerable computational resources, raising concerns about practical deployment in clinical settings. These intensive computational demands may hinder widespread implementation, especially in resource-limited environments (24, 45). These resource demands are closely related to test-time compute, which operationalizes deliberate “slow thinking” by allocating extra inference steps to improve the reliability of reasoning (46). However, this introduces an intrinsic trade-off in latency and operational cost that may conflict with the immediate analytical access needed by clinicians in time-sensitive practice. In addition, DeepSeek-R1 has been reported to experience intense system congestion during operation, and remarkable discrepancies in reasoning time have been observed among models, further complicating real-time use. The tolerance for latency varies significantly across ophthalmic workflows (47). Scenarios such as acute triage, real-time decision-making, and intraoperative support must respond with much speed, whereas tasks like pre-visit planning, report auditing, longitudinal risk stratification, and patient education will be able to afford longer inference times. To address this variance, practical strategies include a tiered response mode providing fast preliminary guidance followed by optional deeper reasoning; precomputation and caching of common scenarios; explicit time-budget controls; and strong human-in-the-loop oversight where rapid decisions are required to ensure safety.

Ethical and regulatory concerns also pose substantial barriers to clinical adoption (48, 49). The protection of patient privacy, data security, and compliance with healthcare regulations remain critical priorities. Because ocular images serve as biometric identifiers, the development of privacy-preserving pipelines—and when appropriate, synthetic ophthalmic media—is essential for secure data sharing and educational purposes. Recent text-to-video systems that convert fluorescence fundus angiography (FFA) reports into dynamic angiography videos have demonstrated realistic retinal findings while maintaining privacy preservation for multicenter use (50). Such approaches provide new inspiration for our own privacy-preserving pipelines.

Chatbot psychosis and unsupervised psychiatric counseling should be framed as a systemic ethical and patient-safety risk, rather than an isolated edge case (43). The central failure mode arises when a generative model is deployed in an open-ended “counseling” format without clinical assessment, risk stratification, and ongoing human oversight, potentially producing confident but inappropriate guidance that can reinforce delusional beliefs, exacerbate anxiety or dependency, and delay timely access to professional care (51). As a mitigation strategy, clinical deployments should preferentially adopt restricted application formats, constraining LLM use to structured psychoeducation, resource navigation, and visit preparation (e.g., organizing concerns and questions) rather than delivering diagnostic or therapeutic psychiatric advice.

Furthermore, addressing biases related to race, sex, and age is imperative to avoid unfair treatment and diagnostic disparities; hence, the necessity for rigorous bias detection and mitigation strategies (52).

Moreover, the transparency and interpretability of models remain essential for clinician trust and practical use (53, 54). Clinicians require understandable explanations of model reasoning processes to effectively integrate AI-driven insights into clinical workflows. For example, during IOL selection, ophthalmic report interpretation, and the generation of pre-and post-operative patient education materials, clinician oversight remains essential. Although human control cannot be eliminated entirely, LLMs have the potential to enable a single clinician to supervise multiple LLMs simultaneously.

In addition, delegating tasks to LLMs introduces distinct ethical challenges. Recent experimental evidence demonstrates that when users (principals) provide high-level goals or example-based prompts rather than explicit rules, LLMs are more prone to exhibit dishonest or ethically questionable behaviors. Moreover, machine agents tend to comply with unethical requests more completely than human agents. Although the addition of strongly worded prohibitive guardrails at the user level has been shown to reduce this tendency, such measures rarely eliminate it entirely. This highlights the importance of explicit and auditable instruction pathways in clinical deployment to safeguard against unsafe compliance (55).

## 6 Conclusion

Development has significantly evolved from simple prompt-engineering methods to more structured inference mechanisms that support deep multi-step reasoning. These changes indicate possible applications in very complex clinical decision-making for medical diagnostics. However, the practical translation to a medical environment will have to be done very cautiously, considering cost, computational efficiency, and safety. Future research should focus on domain-specific medical adaptation, enhancement of reasoning transparency and interpretability, and the development of a regulatory and governance framework specifying various requirements for transparency, auditability, and accountability prior to general clinical use. Importantly, future interventional studies will be needed prior to claims of benefits regarding real-world patient outcomes beyond benchmark or simulated accuracy.
